# Characterization of Morphological, Thermal, and Mechanical Performances and UV Ageing Degradation of Post-Consumer Recycled Polypropylene for Automotive Industries

**DOI:** 10.3390/ma18051090

**Published:** 2025-02-28

**Authors:** Matilde Arese, Beatrice Cavallo, Gabriele Ciaccio, Valentina Brunella

**Affiliations:** 1 Department of Chemistry, University of Turin, 10125 Turin, Italy; matilde.arese@unito.it (M.A.); beatrice.cavallo@unito.it (B.C.); 2 Fiat Research Center SCPA (CRF), Stellantis, 10135 Turin, Italy; gabriele.ciaccio@crf.it

**Keywords:** polyolefin, recycling, sustainability, waste, solar ageing, degradation, talc, carbon black, EPDM, copolymer

## Abstract

Considering the increasing use of plastics in vehicles, the need for sustainable management is becoming a matter of concern. The reintroduction of plastic originated from post-consumer waste in the vehicle manufacturing loop can also be a solution to meet the recent EU ELVs (end-of-life vehicles) legislation in terms of sustainability. This study focuses on post-consumer polypropylene (PP) compounds destined for automotive applications by assessing their morphological, thermal, and mechanical properties. Field Emission Scanning Electron Microscopy (FE-SEM), thermogravimetric analysis (TGA), and differential scanning calorimetry (DSC) techniques were used. Since the ageing of these materials, caused by the thermo-oxidative degradation process, may compromise their performances, a comprehensive study of their behavior, in comparison to the virgin compound counterpart, was necessary to evaluate the fossil replacement possibility. Furthermore, an additional investigation was conducted after subjecting the materials to UV ageing in order to simulate the degradation effect of solar radiation, with the aim of determining the suitability of the recycled materials in long-term applications. In summary, the results support the feasibility of using recycled post-consumer materials mixed with virgin grade in automotive production, highlighting the stability of thermal and mechanical properties, critical for efficient manufacturing. This research underlines the noteworthy progress in the circularity of automotive plastics, providing a sustainable solution for integrating plastic material waste into new vehicle production.

## 1. Introduction

Automobiles have greatly benefited from the use of plastics which have affected the design, functionality, and performance of vehicles [1]. These materials’ use in automobiles can be traced back to the early 20th century, when vulcanized rubber was developed for tires [2]. Since that time, utilization of plastics has risen exponentially, today reaching over 300 kg per vehicle, which represents nearly 20% of the car’s total weight [3]. The primary benefit of incorporating polymers in cars is their ability to lower the overall vehicle weight, therefore contributing to the reduction of CO_2_ emissions, but they also enhance safety and improve comfort and cost-efficiency [4,5]. Consequently, the automotive sector stands as the third largest end-use market for plastics (8.3%), after packaging (39%) and construction (22.9%). Among these materials, PP is one of the most frequently encountered in vehicles. This polyolefin has good mechanical properties and processability, as well as being largely available at relatively low production expenses [6,7]. However, its high glass transition temperature and high crystallinity cause a reduction of toughness at low temperatures [8,9,10]. In order to overcome these drawbacks, elastomers such as ethylene–propylene rubber (EPR), ethylene–propylene–diene terpolymer (EPDM), and styrene–butadiene–styrene (SBS) rubber are added to enhance toughness and impact resistance [11], but this results in reduced yield strength and Young’s modulus values [12,13]. To compensate and complement the effect of the elastomer content, inorganic fillers are added [14,15]. Talc is an economical solution to increase impact energy as well as to improve hardness and to achieve higher modulus and tensile strength [16]. These blends made of PP resin, elastomeric, and inorganic additives are commonly referred to as thermoplastic olefins (TPOs) and are used for a variety of parts in vehicles, including bumpers, instrumental panels, and door trims [12]. Because of the extensive use of plastics, their waste management has become a challenging issue and most of the plastics produced annually end up in the environment [17]. Therefore, strategies to enhance their recovery are urgently needed; for instance, in Europe, only 19.2% of the total resin produced in 2022 derived from circular plastic, meaning over 80% of plastics remain fossil-based [18]. One of the primary weaknesses for such low usage of recycled materials lies in the lack of knowledge of the current recycling approaches and their feasibility. Apart from energy recovery through incineration, plastic recycling primarily follows two approaches: chemical and mechanical recycling. The high energy demands of chemical recycling make mechanical recycling currently preferred, as it offers a more viable pathway for maintaining a closed-loop resources management system [19]. Within mechanical recycling, based on the waste origin, a distinction must be made between post-industrial recycled (PIR) and post-consumer recycled (PCR) products. PIR refers to waste obtained during production and manufacturing before use. This type of waste, which is referred to as by-product, generates high quality products, with a well-known composition and minimal contaminations [20,21]. PCR on the other hand, derives from products after consumer use, often collected as part of a mixed waste stream together with other materials and contaminants. PCR is therefore a mixture of resins with a lower quality compared to PIR, because of the presence of contaminants, the heterogeneity, and the consistent losses during collection, separation, and recycling [22,23]. Despite these drawbacks, PCR has been proven to have a greater impact on the circular economy compared to PIR, as it helps to close the loop at the end of a product’s useful life, enabling materials to be recycled and reused multiple times [24]. Thus, the usage of PCR is essential to achieve a sustainable management of plastics materials, and it has emerged as one of the focal efforts within the automotive sector to minimize its environmental impact. This goal aligns with regulatory mandates like the European Strategy for Plastic in a Circular Economy, which emphasizes recycling and recovery opportunities, particularly in sectors like end-of-life vehicles (ELVs). In that sense, the new draft of the ELV Directive (currently under discussion) orders that by 2035 25% of the plastics used in cars must come from ELV treatment, posing a decisive reform for the disposal of used vehicles. Although around 80% of the ELV’s weight is recycled, the majority of the up-cycle involves metal components, which are rather easy and cost-effective to recover [3]. Because of their varied compositions and lack of financial incentives for recycling, plastics pose a greater challenge in being included in ELV recovery [25]. One of the major barriers to the wider application of recycled plastics is the degradation these materials experience during their lifetime and recycling processes. In fact, polyolefins tend to lose their mechanical properties after severe processing or periods of thermal and light exposure [26]. Many researchers have studied the impact of recycling on the structure and mechanical properties of PP, particularly noticing reductions of the molecular weight and increases of crystallinity, causing higher melt flow rates, higher tensile modulus, and lower elongation at break [2,27,28]. Relatively small chemical change is required to create major changes in physical properties, especially when the material is exposed to weathering in outdoors applications, where degradation is initiated by oxygen and the UV radiation of sunlight [29,30]. Photodegradation starts when chromophore groups, created during the manufacturing process, absorb the UV light, activating free-radical chain reactions. Degradation is mainly concentrated at the surface, where new chemical groups such as carbonyl and hydroperoxide are formed [31]. As a result, the polymer chains become shorter, gaining mobility and facilitating the crystallization, causing the embrittlement of the polymer [32]. In order to contain this phenomenon, UV stabilizers and antioxidants are added [33]; among them carbon black is frequently employed [15,34]. However, due to the loss over time of these stabilizers, it is common practice to add further stabilizers to aged polymers [35,36]. To employ recycled plastics safely in cars and to achieve the EU goals by the end of terms, we must comprehend their behavior and particularly their response to ageing and stress conditions they may encounter during their life cycle. Therefore, this paper studies the changes in mechanical and thermal properties before and after accelerated UV ageing of PP automotive compounds, containing a percentage of post-consumer recycled waste. Although the literature reports a multitude of studies on the behavior of recycled polypropylene, the novelty of this study lies in analyzing how the mechanical and thermal properties of real compounds specifically designed for the automotive sector vary with the incorporation of post-consumer recycled content. Additionally, it examines how such content further affects the degradation of mechanical and thermal properties after aging. This information is of fundamental importance to understand the behavior of these new plastics in the automotive sector, aiming to increase the use of these materials and consequently to enhance sustainability while maintaining aesthetics and safety.

## 2. Materials and Methods

### 2.1. Materials

Four grades of polypropylene–ethylene copolymer compounds applied in the automotive industry were studied. Two samples containing 20% PCR PP, R-PP12 and R-PP20, sourced from end-of-life washing machines, were selected. The virgin counterparts (V-PP12 and V-PP20), composed entirely of fossil-based non-renewable polymers, were used as reference standards. V-PP12 and R-PP12 are designated for interior applications (dashboard, door panel, and central console) while V-PP20 and R-PP20 are destined for exterior applications (bumper). Sample composition is detailed in Table 1. All compounds are impact-modified with EPDM and contain talc as a reinforcing agent, as well as carbon black. Density and melt flow rate (MFR) data were extrapolated from the Technical Data Sheet (TDS).

### 2.2. UV Ageing Test

To simulate solar ageing caused by UV radiation, the Q-SUN Xe-2 Test Chamber was used. The irradiation source consists of a xenon-arc lamp equipped with quartz–boron filters with an irradiance setting of 0.55 W/m^2^ at 340 nm. Considering the different final applications of the samples on vehicles, they were subjected to distinct levels of radiant exposure: samples 1 and 2, intended for interior applications, were irradiated with 225 kJ/m^2^, 601 kJ/m^2^, and 1240 kJ/m^2^, while samples 3 and 4, designed for exterior use, were exposed to 1250 kJ/m^2^, 1800 kJ/m^2^, and 2500 kJ/m^2^. Characterization of all compounds was performed before and after each aging phase to investigate their resistance to photodegradation over time.

### 2.3. FE-SEM Analysis

Morphological characterization of the samples was performed by Field Emission Scanning Electron Microscopy (FE-SEM) TESCAN S9000G (TESCAN ORSAY HOLDING, Brno, Czechia) equipped with an Oxford Ultim-Max energy-dispersive X-ray spectroscopy (EDS) system (Oxford Instruments, Abingdon, UK), with AZtec software (Casali del Manco (CS), Italy) version AztecLive Advance 6.1 SP2, operating at 10.00 keV. The samples were firstly cryogenically fractured in liquid nitrogen and metalized before analysis with 12 nm gold layer using a VAC COAT DSR sputter coater (London, UK). PP materials were additionally studied after RuO_4_ staining. The rubber phase was then stained with ruthenium tetroxide (RuO_4_) vapor to increase the phase contrast in backscattering electron (BSE)-SEM mode. RuO_4_ vapors were produced by mixing RuO_2_ hydrate with an aqueous solution of NaIO_4_. A staining time of 2.5 h at 5 °C was used. To remove overstained materials the samples were dipped into 1% aqueous solution of NaIO_4_. For this phase of the analysis, samples were coated with a 6 nm gold layer and examined at 15 keV.

### 2.4. Thermal Analysis

Thermal stability and fillers content were measured through thermogravimetric analysis (TGA), using a TGA Q500 brand TA Instrument (New Castle, DE, USA). Approximately 10 mg of sample was placed in an alumina pan and heated at a rate of 10 °C/min under an air flow of 60 mL/min. The analysis was conducted in a nitrogen atmosphere from 50 to 700 °C, then switched to oxygen until 800 °C. Three measurements per sample were performed. Differential scanning calorimetry (DSC) was conducted on a TA Instrument DSC Q200 equipment (New Castle, DE, USA) to investigate melting and crystallization temperature. Approximately 6 mg of sample was placed in a hermetic aluminum pan and heated under an inert nitrogen atmosphere with a flow rate of 50 mL/min from 10 °C to 190 °C at a heating and cooling rate of 10 °C/min. A secondary analysis was performed at 20 °C/min to enhance the detection of an additional peak during the first heating. The melting temperature Tm and enthalpy of fusion (∆Hm) were obtained from the first heating ramp. The crystallization temperature Tc was taken from the cooling ramp [7,26]. The melting enthalpy (∆Hm) was calculated by integrating the melting peak within the temperature ranges of 130 °C to 180 °C. Each PP sample was analyzed in triplicate to ensure reproducibility.

### 2.5. Fourier Transform Infrared (FT-IR) Analysis

Fourier transform infrared spectroscopy (FTIR) was run on a Perkin Elmer Spectrum 100 FT-IR spectrophotometer (Waltham, MA, USA) with the aim of examining the presence of chemical reactions within the structure of samples. The test was conducted in attenuated total reflection (ATR) mode equipped with an internal diamond reflection element. All the spectra were acquired in absorbance, using 16 scans between 4000 cm^−1^ and 650 cm^−1^ wavenumber range and a resolution of 4 cm^−1^.

### 2.6. Impact Behavior Test

The Izod impact test was achieved with a CEAST Resil Impact Junior pendulum brand Instron (Darmstadt, Germany) according to ISO 180/A [37]. The impact of the specimens occurred through a striker of 2 J energy, in a controlled atmosphere at 23 °C and 55% of humidity. The final value was recorded from an average of seven standard specimens obtained by injection molding. The ASTM D3763 standard [38], which regulates the investigation of high-speed puncture properties of plastic, was applied to evaluate the multiaxial impact properties of the PP specimens at various temperatures. The instrument setup is made by a clamp assembly constituting two circular parallel plates with a 76 mm diameter hole in the center and a cylindrical 12.7 mm diameter steel striker with a hemispherical shape centered on the clamp hole. During impact, the striker moves down and the polymer plaque, clamped by the circular plates, is totally penetrated. The objective is to evaluate the energy absorbed by the plate through a force transducer inside the dart and measuring its displacement over time. This energy is analogous to how the polymer deforms and fractures due to its strain-rate dependent elastic and plastic behavior. The total energy absorbed is the sum of the spring-like elastic components and permanent plastic deformation. The test was conducted in a controlled atmosphere with 55% relative humidity across a range of temperatures (23 °C, 0 °C, −10 °C, −20 °C, and −30 °C) using a CEAST 9350 drop tower, Instron brand (Darmstadt, Germany equipped with a 22 kN TUP strain gauge. For interior materials, the test was performed with a striker speed of 6.6 m/s and a mass of 10 kg, while for exterior materials, a speed of 2.2 m/s and a mass of 65 kg were used. The results were averaged from 10 specimens of 90 × 90 × 3.2 mm dimension obtained by injection molding.

### 2.7. Mechanical Characterization

Tensile properties were measured in accordance with ISO 527 [39], using an Alliance RF/100 dynamometer brand MTS Systems Corporation (Eden Prairie, MN, USA) equipped with a 10 kN load cell. The test was conducted by applying a tensile force at a controlled speed of 1 mm/min during the elastic region and 50 mm/min in the plastic region. Testing took place in a controlled environment at 23 °C and 55% relative humidity, and the final values were calculated as the average from seven ISO 527 (Type 1A) specimens obtained by injection molding. The flexural test, used to determine the flexural modulus, was performed on the same Alliance RF/100 dynamometer, also equipped with a 10 kN load cell. Following ISO 178 [40] guidelines, the test was carried out at a strain rate of 1%/min, corresponding to a speed of 2 mm/min, under the same controlled conditions (23 °C, 55% humidity). The final flexural modulus was derived from the average of seven ISO 178 specimens obtained by injection molding.

## 3. Results

### 3.1. FE-SEM

**Figure 1:**
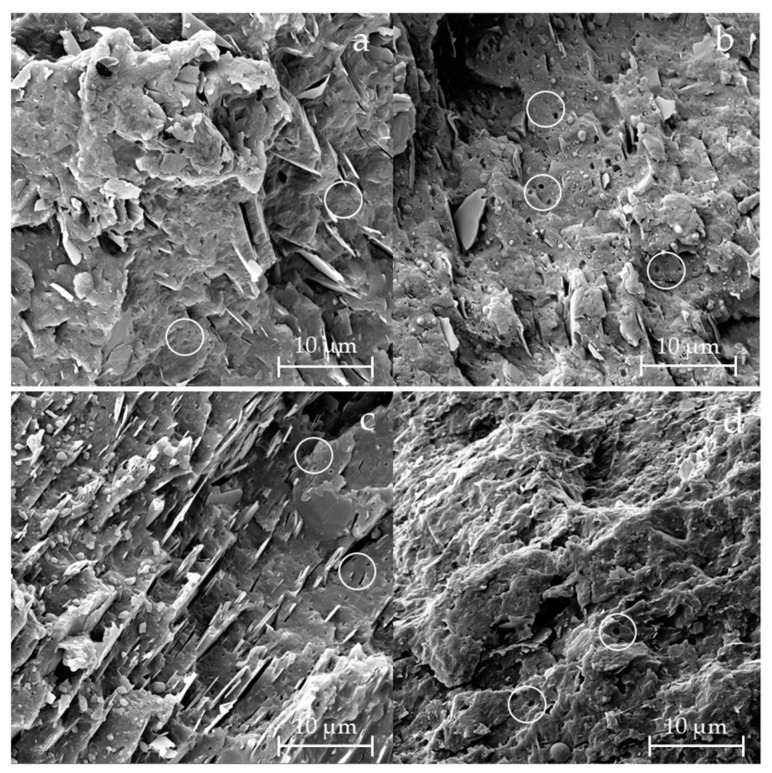
FE-SEM images of samples V-PP12 (**a**), R-PP12 (**b**), V-PP20 (**c**), and R-PP20 (**d**) after cryofracture in nitrogen, using SE detection. The white circles shown in the images were added to highlight the presence of small circular holes, which confirm the presence of an elastomeric phase within the polymer matrix.

**Figure 2:**
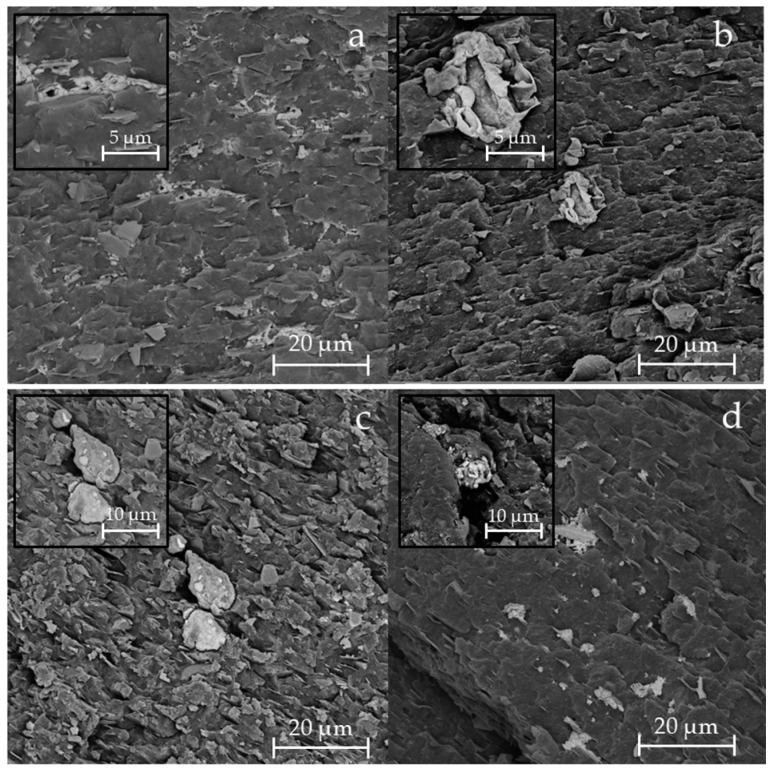
FE-SEM images of samples V-PP12 (**a**), R-PP12 (**b**), V-PP20 (**c**), and R-PP20 (**d**) after cryofracture in nitrogen, using BSE detection.

To better understand the differences in morphology between virgin and recycled samples, SEM analysis was performed on cryo-fractured surfaces of the materials (Figure 1). Comparing V-PP12 with R-PP12, the surface of the virgin samples appeared more brittle, while R-PP12 exhibited a more deformed surface, compatible with a higher content of elastomeric phase. A similar trend was observed for V-PP20 and R-PP20, where the recycled samples showed greater plastic deformation compared to its virgin counterpart [41]. In all samples, but particularly evident in the virgin materials Figure 1a,c, characteristic talc lamellae were clearly visible and oriented in the flow direction [42]. The presence of elastomeric phase, such as EPDM, was confirmed by the presence of small circular holes in the polymer matrix, highlighted in Figure 1 [25]. These domains appeared as globular structures, indicative of rubber domain cavitated from the continuous polymer matrix [43]. According to Tang et al. [44], when the EPDM content is up to 50 wt.%, EPDM phase and PP matrix form a co-continuous morphology, which can be considered as a three-dimensional (3D) structure that consists of elongated interconnected domains. In our case, instead, was it possible to discriminate clear round holes, typical of an elastomeric phase amount below 30 wt.%. To further confirm the presence of the elastomeric phase, FE-SEM analysis was performed after staining in ruthenium tetroxide. The contrast between different polymer components can be enhanced by preferential chemical staining with a staining agent (such as heavy metals), which increases the electron density of one of the components [45]. In Figure 2, the elastomeric phase appeared white, while the PP matrix was the dark one [46]. The large atomic number of Ru gives it a high backscatter coefficient, creating this contrast [47]. This analysis confirmed the dispersion of elastomeric phase within the PP matrix [48].

### 3.2. Thermal Analysis

**Figure 3:**
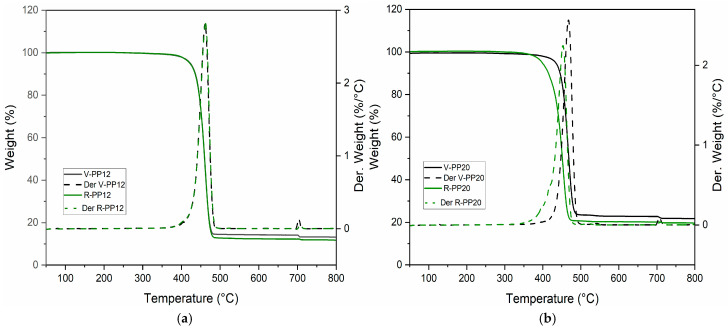
(**a**) TG and DTG of V-PP12 and R-PP12 before ageing (**b**) TG and DTG of V-PP20 and R-PP20 before ageing.

**Figure 4:**
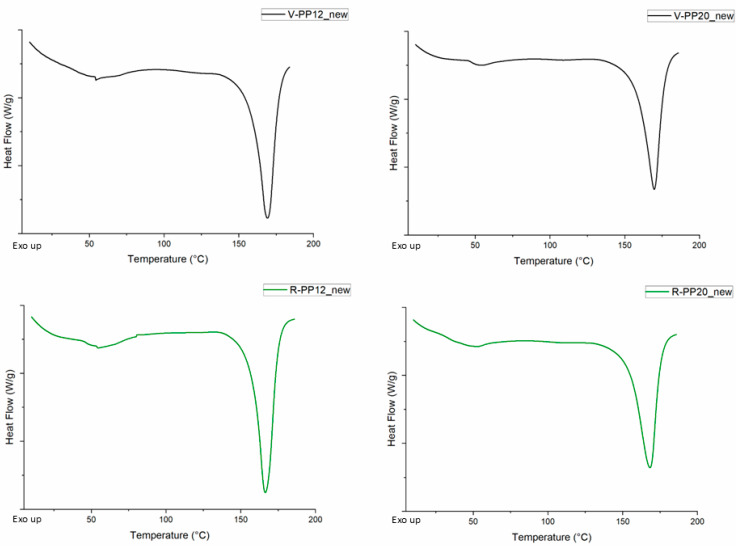
First heating melting peaks of samples V-PP12, R-PP12, V-PP20, and R-PP29 before ageing. Thermograms were acquired at 20 °C/min.

The TGA results are collected in Table 2, which reports the mean values and standard deviations obtained from three repeated measurements of each sample; additional values are reported in the Supplementary Materials. TG and DTG of all samples are shown in Figure 3. The organic phase related to the polymer backbone degradation of PP and elastomeric components was derived by calculating the difference between the initial weight and the weight of the residue at 500 °C. An additional loss was registered at 700 °C, in correspondence with the switch in oxidative atmosphere, identified as oxidable carbonaceous fillers, like carbon black. In each sample, a residue at 800 °C was detected, attributable to the presence of an inorganic filler. TG analyses also indicated that the temperature of start degradation (T onset) of virgin PP was higher compared to the recycled ones. This phenomenon could be attributed to a reduction in the polymer chain length caused by the recycling process, which leads to the release of volatile products at lower temperatures, resulting in an earlier onset of degradation [49,50]. Another relevant difference was the carbon black content, which resulted in being higher in virgin samples. Table 3 illustrates the data extrapolated from DSC thermograms including peak temperatures and enthalpy values for melting events of virgin and recycled PPs before and after the last step of UV ageing (relative thermograms are reported in the Supplementary Materials). All materials presented an intense peak around 166 °C, corresponding to melting temperature of isotactic PP typically used for impact application [51,52]. Additionally, a broad, low-intensity endothermic peak around 54 °C was observed in the first heating cycle (Figure 4) for all four samples. This signal is associated with the presence of dispersed elastomeric phase that is commonly added to component subjected to impact (e.g., dashboard and bumpers) to improve flexibility and toughness, such as EPDM (ethylene–propylene–diene monomer), as supported by previous studies [25] and also demonstrated by FE-SEM analysis. No differences in melting and crystallization temperature were evident between virgin and recycled samples, even after UV aging. This stability can be attributed to the presence of fillers such as talc and carbon black, which have the capability of absorbing more heat energy in the melting [20]. The decrease in the melting enthalpy of samples with 20% talc is due to the reduction in the crystalline fraction of PP [53]. Since the ΔHm is proportional to the amount of crystalline fraction, the addition of talc, which does not participate in the crystallization process, leads to a reduction in this value. Moreover, the increase in talc content may reduce the mobility of polymer chains during cooling, thereby decreasing crystalline order and, consequently, the melting enthalpy [54].

### 3.3. FT-IR

**Figure 5:**
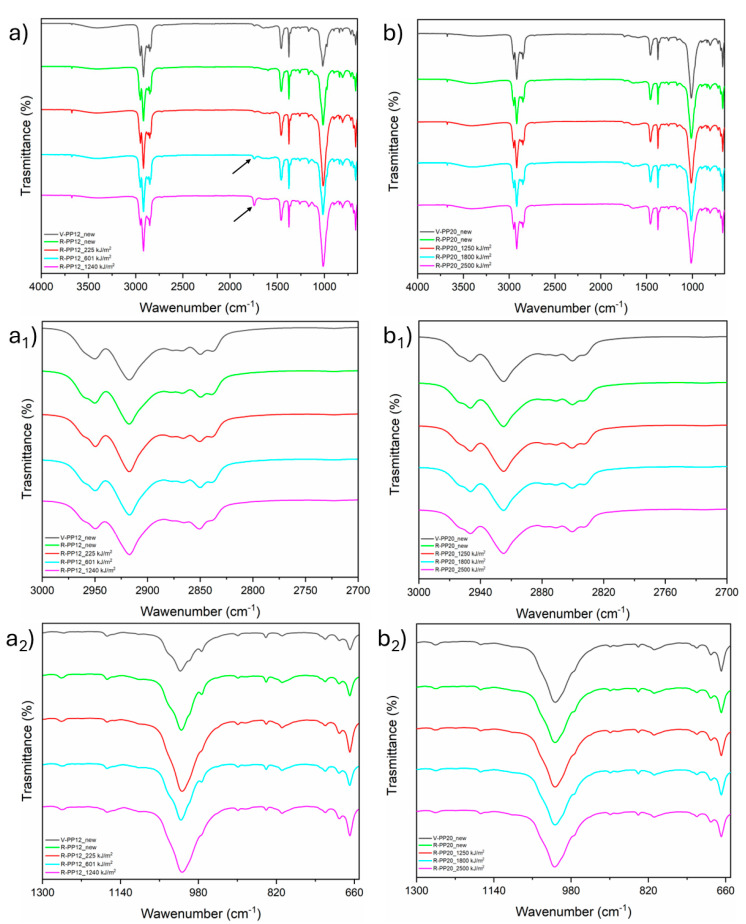
Graphical illustration of ATR-FTIR transmission spectra of: (**a**) entire wavenumber range investigated of V-PP12 before ageing and R-PP12 after all ageing steps, (**a_1_**) V-PP12 and R-PP12 magnification of CH2 and CH3 stretching vibration region, (**a_2_**) V-PP12 and R-PP12 magnification of bending and skeletal vibration region; (**b**) entire wavenumber range investigated of V-PP20 before ageing and R-PP20 after all ageing steps, (**b_1_**) V-PP20 and R-PP20 magnification of CH2 and CH3 stretching vibration region, (**b_2_**) V-PP20 and R-PP20 magnification of bending and skeletal vibration region.

The FT-IR spectra of V-PP12 and V-PP20 before ageing and R-PP12 and R-PP20 before and after the three ageing steps are shown in Figure 5. All spectra were quite similar, displaying the characteristic peaks of PP [55]. A broad, flat peak between 3600 cm^−1^ and 3100 cm^−1^ was observed in all samples. Such broad IR bands above 3000 cm^−1^ are often attributed to hydroxyl groups and hydrogen bonds, which may originate from diverse impurities [56], including associated hydrogen bonds of surface-bound water. This presence is likely due to hygroscopic additives or polar contaminants on the sample surface [57]. A band around 2950 cm^−1^ corresponds to the asymmetrical stretching vibrations of CH_3_ on the PP surface [58]. Observing the C-H stretching vibration region, two peaks at 2850 cm^−1^ and 2838 cm^−1^ were noticed. Both bands correspond to the symmetric C-H stretching vibration of the CH_2_ groups, but the one at 2850 cm^−1^ is characteristic of PE while the band at 2838 cm^−1^ occurs in PP [56]. A carbonyl peak at 1745 cm^−^^1^ was present in all PP spectra. This carbonyl functionality in polyolefins may arise from thermo-oxidative or photochemical degradation, as well as from the presence of additives based on esters. No significant changes were observed in the carbonyl peak for R-PP20 after ageing (Figure 5b), while a slight increase was detected in R-PP12 after 601 kJ/m^2^ and 1240 kJ/m^2^ irradiation (Figure 5a). These results provide evidence that neither the virgin nor the recycled samples underwent significant degradation due to UV irradiation. The spectra also showed characteristic peaks of partially crystalline PP. Peaks at approximately 1165, 972, and 843 cm^−1^ correspond to asymmetrical C-H stretching vibration, CH_3_C-C rocking, and other crystalline-related vibrations, respectively. In particular, the shoulder at 974 cm^−1^ is attributed to both amorphous and crystalline regions of the polymer [59]. Both the 1165 and 841 cm^−1^ bands belong to the group of regularity bands, and they are related to different helix lengths of isotactic sequences [60] and are generally used to monitor the crystallization kinetics of isotactic polypropylene (iPP) [61]. A twin peak at 730/720 cm^−^^1^ was visible in all PP spectra. This pattern, typically found in PE materials, can be found in PP copolymers, but the intensity is typically very low due to low molar concentration of ethylene units. This confirms the copolymer nature of all PP compounds investigated in this study. The detection of this twin peak is hence in agreement with the pattern at 2850/2838 cm^−1^ described above [56]. The presence of talc was confirmed by a band at 670 cm^−1^ [62]. Additionally, two other talc-related bands were detected: a high-intensity band at 1018 cm^−1^ (Mg_3_-OH stretching vibration) and a low-intensity band at 3676 cm^−1^ (Si-O-Si stretching vibration) [63].

### 3.4. Impact Test Analysis

**Figure 6:**
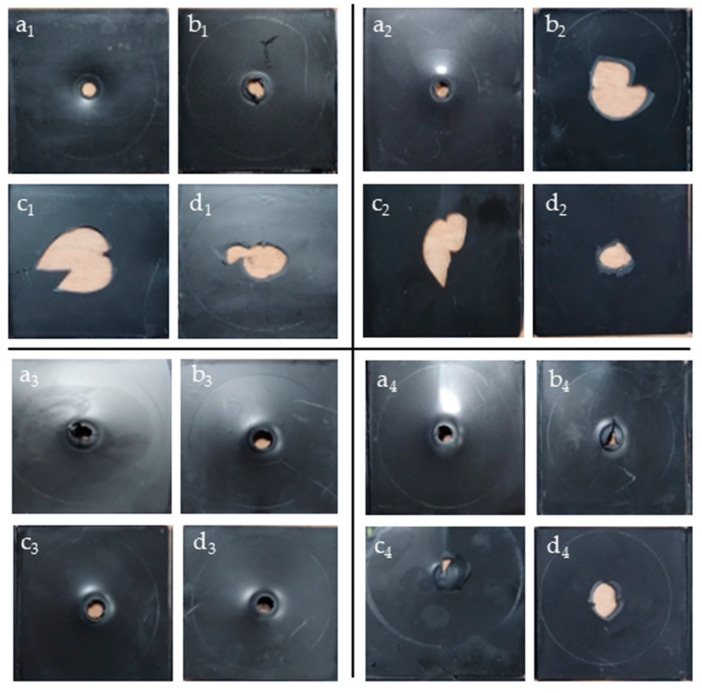
Breakage results after multiaxial impact test at different temperatures: V-PP12 at 23 °C (**a_1_**), V-PP12 at −10 °C (**b_1_**), V-PP12 at −20 °C (**c_1_**), V-PP12 at −30 °C (**d_1_**); R-PP12 at 23 °C (**a_2_**), R-PP12 at −10 °C (**b_2_**), R-PP12 at −20 °C (**c_2_**), R-PP12 at −30 °C (**d_2_**); V-PP20 at 23 °C (**a_3_**), V-PP20 at −10 °C (**b_3_**), V-PP20 at −20 °C (**c_3_**), V-PP20 at −30 °C (**d_3_**); R-PP20 at 23 °C (**a_4_**), R-PP20 at −10 °C (**b_4_**), R-PP20 at −20 °C (**c_4_**), R-PP20 at −30 °C (**d_4_**).

**Figure 7:**
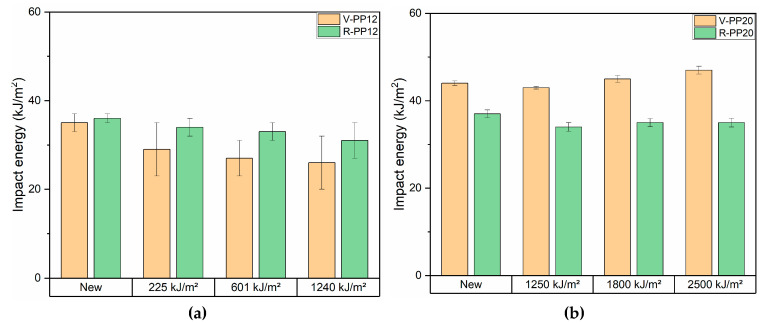
Impact energy results before and after ageing of samples V-PP12 and R-PP12 (**a**) and V-PP20 and R-PP20 (**b**).

The multiaxial impact test was executed on samples before ageing to evaluate the ductile-to-brittle transition at low temperature. In the automotive industry, it is crucial to determine the ductile and brittle behavior of plastic materials, especially those intended for safety components (e.g., dashboard, door panel and bumpers). The material’s performance was assessed through optical analysis of the fracture surface after impact and study of the force–deflection curves. Table 4 summarizes the results of multiaxial impact test conducted at 23 °C, −10 °C, −20 °C, and −30 °C for all samples before ageing, while Figure 6 displays the images of the fracture. At 23 °C, all materials exhibited a ductile deformation characterized by a pronounced plastic deformation during crack propagation. However, differences in behavior became apparent at lower temperatures. Considering PP12 samples, the virgin PP presented a brittle fracture at −20 °C, whereas the recycled one exhibited brittle behavior as early as −10 °C. The brittle behavior was identified by the fracture morphology, recognizable as the hole left by the striker is characterized by cracks and sharp edges. The same trend was observed for PP20s: the virgin PP maintained ductile behavior down to −30 °C, while the recycled one transitioned to brittle at −20 °C. The V-PP12 sample exhibited a ductile-to-brittle transition at lower temperatures compared to its recycled counterpart. This highlights that the recycling process and the associated chain scission result in reduced impact performance at low temperatures. A similar phenomenon was observed in the samples containing 20% talc. Comparing the results of the V-PP12 and V-PP20 samples, the fact that the sample with 20% talc remains ductile at −30 °C is attributed to the reinforcing effect of talc, present in higher amounts than in V-PP12, combined with the presence of the elastomeric fraction. Indeed, the impact strength of a PP material can be enhanced by adding talc only when the polymer is blended with an elastomeric material [64]. Figure 7 illustrates the impact strength of samples V-PP12, R-PP12, V-PP20, and R-PP20 before and after UV aging. For PP12 samples, both virgin and recycled PP exhibited comparable impact values before ageing. The differences emerged after the first step of ageing, where recycled samples showed superior performance. Indeed, a low decrease of impact energy is registered for the R-PP12 compared to the significant reduction observed in V-PP12. This difference has been already investigated in previous studies, in which, in some cases, virgin materials were shown to be the most damaged samples regarding thermos-oxidation degradation [23]. About PP20s, an inverted trend was observed: the virgin sample exhibited higher impact strength values, with an initial decrease in performance after the initial ageing step, followed by a maintenance of values in the last two [65]. These last results were in accordance with the multiaxial impact findings, which demonstrated superior ductile behavior in V-PP20 under impact conditions.

### 3.5. Mechanical Properties

**Figure 8:**
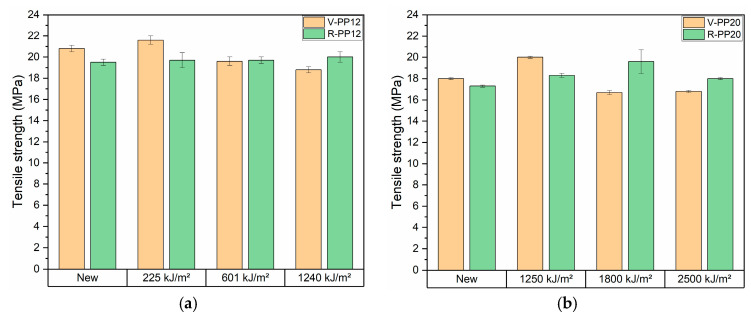
Tensile test results before and after ageing of samples V-PP12 and R-PP12 (**a**) and V-PP20 and R-PP20 (**b**).

**Figure 9:**
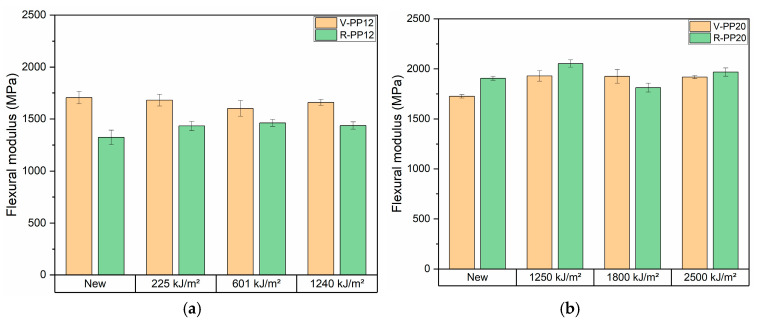
Flexural test results before and after ageing of samples V-PP12 and R-PP12 (**a**) and V-PP20 and R-PP20 (**b**).

The tensile test results are presented in Figure 8 for PP12 and PP20, before and after ageing. Initially and in the first step of ageing, V-PP12 possessed a higher strength at break compared to R-PP12. Then, V-PP12 exhibited a decline in strength in the final two aging steps, while R-PP12 maintained a stable value throughout all aging phases. About the PP20 samples, both virgin and recycled PP showed comparable values before and after ageing. A slight increase in tensile strength was registered for V-PP20 at 1250 kJ/m^2^, followed by stabilization in ageing time, R-PP20 maintains stability over all the ageing. Flexural test results (Figure 9) showed a lower flexural modulus for RPP12 compared to VPP12. Despite this difference, V-PP12 exhibited stable values during time, while R-PP12 experienced an increase in flexural modulus during the first stage of ageing, followed by a maintenance of the values over time. For the PP20 samples, recycled PP demonstrated slightly higher flexural modulus values respect the virgin version. V-PP20 showed an increase of flexural modulus at 1250 kJ/m^2^, which then remained stable throughout the aging process. R-PP20 experienced a similar trend, with stable values over time. These findings align with previous studies showing that PCR and virgin materials exhibit similar mechanical behavior, with only minor differences in flexural modulus. Eventually, it can be stated that the polymer matrix was minimally affected by the oxidation process caused by UV irradiation and retained its mechanical properties over time. This is further confirmed by the IR analysis, which showed no significant increase in the oxidation band [25].

## 4. Conclusions

This study demonstrated the potential of utilizing PCR-PP compounds as an alternative to virgin PP in automotive applications, considering the increasing regulatory pressures and sustainability goals the automotive sector is currently facing. In summary, the results support the feasibility of using recycled post-consumer materials mixed with virgin grade in automotive production, highlighting the stability of thermal and mechanical properties critical for efficient manufacturing. However, a limitation was observed in both types of recycled PP studied regarding the multiaxial impact behavior at low temperature, as well as a slight reduction in flexural modulus. On the other hand, good performances in terms of aging stability, especially when subjected to UV radiation, were registered. Moreover, the SEM analysis highlighted the importance of understanding the material morphology for a comprehensive interpretation of the mechanical properties of these polymeric materials. Given the growing importance of circularity in the automotive sector, this research reinforced the importance of post-consumer recycles as a means to close the material loop, and demonstrated that is possible to obtain a performant materials replacing 20% of virgin resin with a recycled blend from post-consumer waste. These materials, in addition, retained their properties even after undergoing multiple cycles of accelerated aging simulations. This supports the potential for using post-consumer recycled PP in applications requiring high performance, such as automotive components, establishing an open-loop recycling process and thus promoting a new economy based on industrial ecology.

## Figures and Tables

**Table 1:** Sample summary information.

Sample	Material Code	Talc (%)	PIR (wt.%)	Density (g/cm^3^)	MFR (g/10 min)
1	V-PP12	12	–	0.97	30
2	R-PP12	12	20	0.98	30
3	V-PP20	20	–	1.04	22
4	R-PP20	20	20	1.04	30

**Table 2:** TGA analysis results of virgin and recycled samples before ageing.

Sample	T Onset	% Polymer [*w*/*w*]	% CB [*w*/*w*]	% Residue [*w*/*w*]
V-PP12	425 ± 2.2	86 ± 0.1	0.9 ± 0.04	13 ± 0.2
R-PP12	411 ± 15.7	87 ± 0.71	0.5 ± 0.02	12 ± 0.6
V-PP20	429 ± 9.5	78 ± 0.05	0.9 ± 0.41	22 ± 0.4
R-PP20	417 ± 15.5	80 ± 3.9	0.5 ± 0.2	19 ± 3.9

**Table 3:** Mean values of melting peak temperature Tm, melting enthalpy ∆Hm, and crystallization peak temperature Tc (reported along with the corresponding standard deviations) derived from DSC before and after the last step of ageing (1240 kJ/m^2^ for sample 1 and 2 and 2500 kJ/m^2^ for sample 3 and 4).

Sample	Tm (°C)	Tc (°C)	Δhm (J/g)
Before ageing			
V-PP12	166 ± 0.5	128 ± 0.1	53 ± 1.9
R-PP12	167 ± 0.5	127 ± 0.5	54 ± 1.9
V-PP20	166 ± 0.6	129 ± 0.1	44 ± 2.2
R-PP20	166 ± 0.1	129 ± 0.6	47 ± 1.6
After III ageing step			
V-PP12	166 ± 0.6	128 ± 0.6	56 ± 1.4
R-PP12	166 ± 0.6	129 ± 0.1	49 ± 0.3
V-PP20	167 ± 0.6	129 ± 0.1	43 ± 0.6
R-PP20	166 ± 0.6	128 ± 0.5	47 ± 0.8

**Table 4:** For each sample is reported at what temperature they show ductile break and in what range of temperature ductile-to-brittle break transition occurs.

Sample	Ductile Break	Ductile/Brittle Transition
V-PP12	−10 °C	−10 °C/−20 °C
R-PP12	0 °C	0 °C/−10 °C
V-PP20	−30 °C	>−30 °C
R-PP20	−10 °C	−10 °C/−20 °C

## Data Availability

The original contributions presented in the study are included in the article and Supplementary Materials; further inquiries can be directed to the corresponding authors.

## Supplementary Materials

The following supporting information can be downloaded at: https://www.mdpi.com/article/10.3390/ma18051090/s1, Table S1. Thermal stability values of V-PP20, R-PP20, V-PP12, and R-PP12 calculated at 0.5%, 2.5%, and 5% weight loss and Td max values. Figure S1: Second heating melting peaks of samples V-PP12, R-PP12, V-PP20, and R-PP29 before ageing and after ageing. Thermograms were acquired at 10 °C/min.
