# Supplementary material for: Characterization of Morphological, Thermal, and Mechanical Performances and UV Ageing Degradation of Post-Consumer Recycled Polypropylene for Automotive Industries
Source: Materials (Basel). 2025 Feb 28;18(5):1090. doi: 10.3390/ma18051090 (PMC11901271; doi:10.3390/ma18051090)
Supplement: Supplementary file 1 [file materials-18-01090-s001.zip › materials-3403231-supplementary.pdf]

materials

MDPI

Supplementary Materials

Characterization of Morphological, Thermal, and Mechanical Performances and UV Ageing Degradation of Post-Consumer Recycled Polypropylene for Automotive Industries

Matilde Arese [1,2], Beatrice Cavallo [1,2], Gabriele Ciaccio [2] and Valentina Brunella [1,*]

[1] Department of Chemistry, University of Turin, 10125 Turin, Italy; matilde.arese@unito.it (M.A.); beatrice.cavallo@unito.it (B.C.)
[2] Fiat Research Center SCPA (CRF), Stellantis, 10135 Turin, Italy; gabriele.ciaccio@crf.it
* Correspondence: valentina.brunella@unito.it; Tel.: +39-011-6707546

Table S1. Thermal stability values of V-PP20, R-PP20, V-PP12 and R-PP12 calculated at 0.5%, 2.5% and 5% weight loss and Td max values.

Sample	Td 0.5%	Td 2.5%	Td 5%	Td max
V-PP12	378	405	423	461
R-PP12	337	405	422	462
V-PP20	372	425	438	467
R-PP20	348	383	398	453

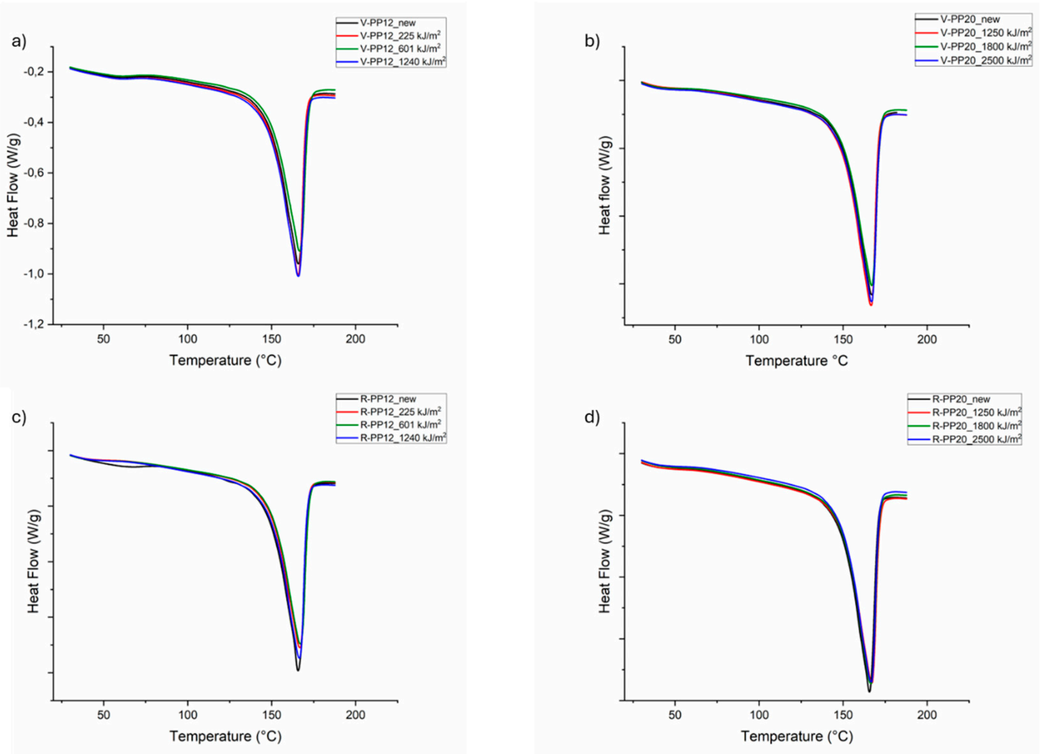

Figure S1. Second heating melting peaks of samples V-PP12, R-PP12, V-PP20 and R-PP29 before ageing and after ageing. The thermograms were acquired at 10°C/min.
